# Unaddressed regulatory issues in xenotransplantation: a hypothetical example

**DOI:** 10.3389/frtra.2023.1222031

**Published:** 2023-09-20

**Authors:** Koko Kwisda

**Affiliations:** Centre for Ethics and Law in the Life Sciences, Leibniz University Hannover, Hanover, Germany

**Keywords:** transplantation, organ shortage, regulation, xenotransplantation, interspecies blastocyst complementation, genome editing technologies, transgenic pigs, biotechnology

## Abstract

The last few years have seen a significant increase in the use of technology to manipulate genetic sequences and generate animals as a source of xeno-organs. This has made the generation of genetically bespoke organisms a reality. This paper will analyze the regulatory and practical aspects of such an innovative approach to xenotransplantation on the basis of a hypothetical case study applied to Germany and highlight the gaps in the current regulation. This paper thus provides the basis for legal debate within a specific country. In addition, the identified gaps also pose a barrier toward the harmonization of international regulation. This publication therefore lays the groundwork for guiding the international debate regarding the regulatory framework for solid organ xenotransplantation toward specific issues.

## Introduction

The shortage of human donor organs is a global problem. Emerging xenotransplantation approaches suggest two possible solutions: genetic modification of the porcine genome to yield organs from these multitransgenic pigs, which are immune-compatible with humans (reviewed in (1–3)); or the introduction of human induced pluripotent stem cells (iPSCs) into pigs to rescue organogenesis of a previously knocked-out target organ via blastocyst complementation: the resulting chimeric animal would ideally have an organ made up of human cells, which would enable proper physiological functions in the recipient's organism (reviewed in (4, 5)).

There are still some obstacles to overcome regarding immune-compatibility and the long-term survival of porcine organs. However, a pig xenograft has been able to survive over 900 days in primates already (6). Notably, the most recent successes were a pig kidney being transplanted into a human, brain-dead recipient, which remained functional for roughly 3 days before the experiment was terminated (7), as well as the first pig to human cardiac xenotransplantation, with the patient surviving for 2 months (8).

In general, multitransgenic pigs could become a seemingly unlimited source of organs. This could have several beneficial effects (reviewed in (9)), namely, avoiding deaths of patients waiting on the transplant list; avoiding considerable costs for managing end-stage care, e.g., for end-stage kidney disease (10); and expanding indications for organ transplantation by including patients ineligible for transplantation per the current standards. The latter would include patients not sick enough or too sick to be eligible according to the current criteria because an unlimited pool of donors would allow individual assessment of every patient because he or she would not be competing for an organ.

Xenotransplantation via blastocyst complementation would most likely be a complementary approach to multitransgenic pigs, as the latter would be the ideal host animals into which to insert the human iPSCs. However, xenotransplantation of a chimeric organ offers several additional possibilities over xeno-organs from “just” multitransgenic pigs (reviewed in (9)):

• *Avoiding immunosuppression*: as the organ would consist of the patient's own cells, immunosuppression could potentially be avoided, or at least limited.
• *Correcting genetic defects*: the iPSCs from the patient could be edited before inserting them into the pig blastocyst and thus correct defective genes. This could be beneficial for patients with genetically determined organ diseases, such as polycystic kidney disease, hemochromatosis, arrhythmogenic cardiomyopathy, cardiac channelopathies, or X-linked chronic granulomatous disease.
• *Compensating for human-specific organ needs*: specific organs or cells are not easily replaceable by their porcine counterparts. This is particularly true for pancreatic islet cells as their insulin secretion capacity apparently does not mirror the human demand for insulin (11); and the liver, as it plays a central role in the production of roughly 2,000 proteins and it seems unlikely that all of those produced by a transplanted porcine liver would function properly in humans (12).

**Figure 1:**
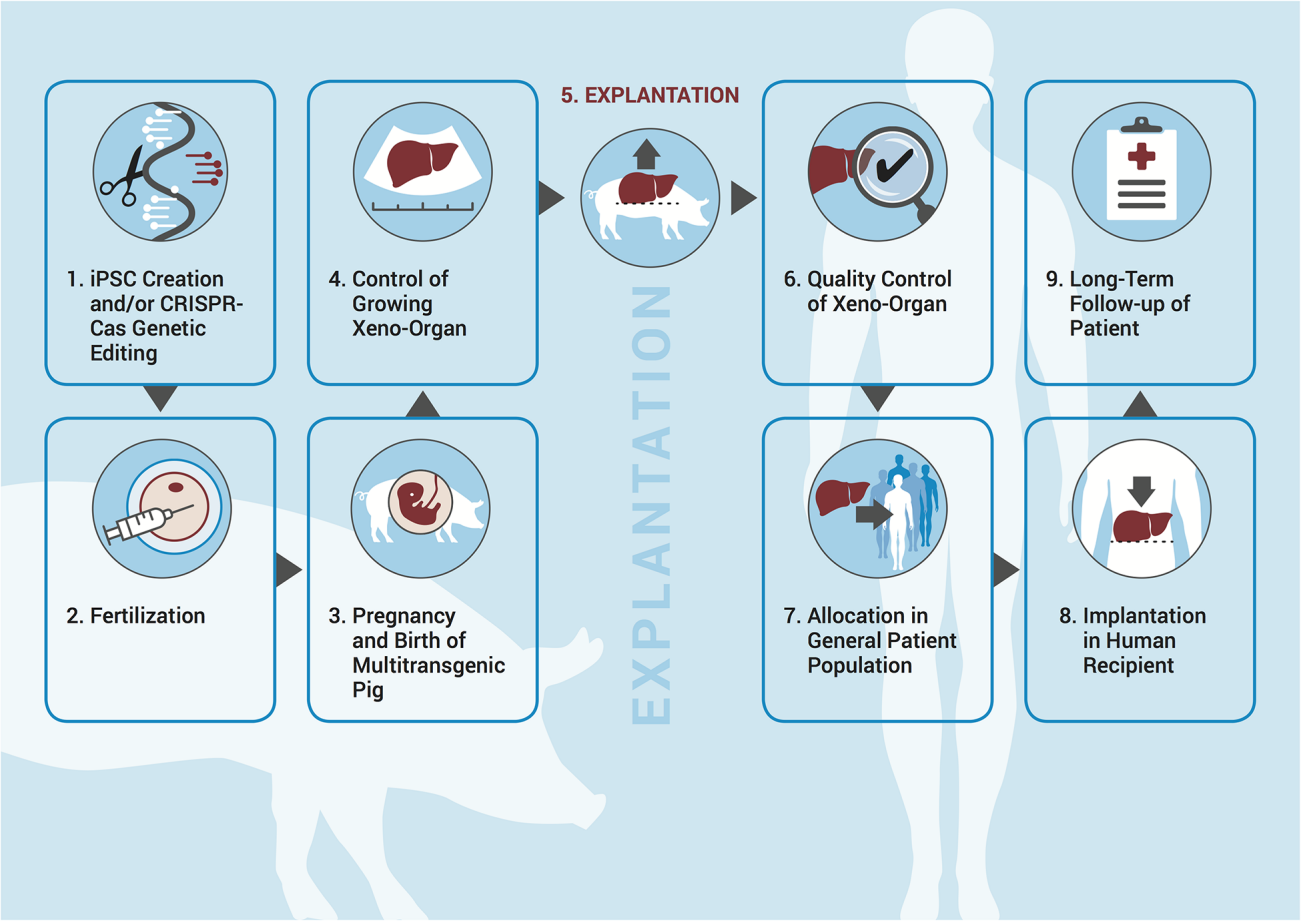
Necessary steps in the process of creating human–animal chimeras/genetically altered pigs for the purposes of xenotransplantation.

It is clear that numerous regulatory concerns need to be addressed before such an approach can be translated into routine practice. In our previous work, we described three key issues pertaining to the creation of human–pig chimeras/multitransgenic pigs, namely: (1) the potential uncertainty as to which framework captures human–animal chimeras or multitransgenic pigs; (2) what the end product is and by which regulation it is captured; and (3) who the owner of the xenoproduct is (13, 14). While we previously discussed these in terms of hindrances to prospective supranational or international regulatory frameworks, we will now play out the scenario in a concrete jurisdiction by describing the necessary steps to be undertaken in order for a patient to receive a chimeric organ for two reasons. First, the science pertaining to multitransgenic pigs is much advanced if compared to blastocyst complementation and all recent successes described above were achieved with transplanting xeno-organs from multitransgenic pigs. Acknowledging that, two important supranational bodies, i.e., the World Health Organization and the International Xenotransplantation Association, have published their own guidelines in an effort to harmonize the approach to xenotransplantation (15–17). Second, human stem cell research is very differently regulated even within EU member states, and mixing them with animal material adds another intriguing level of complexity. Therefore, based on a hypothetical but realistic case study, we will outline the major associated normative and practical issues as described in Figure 1 and address the abovementioned issues in the context of the German jurisdiction.

### The hypothetical case: chimeric humanized organs as an alternative to liver transplant

A 35-year-old patient is diagnosed with intermediate stage hepatocellular carcinoma (HCC) consisting of two separate nodules with sizes of 4 and 1.5 cm, respectively. Subsequent tumor staging analyses confirm the absence of extrahepatic tumor manifestations and no affections of larger branches of the liver vasculature are detectable. The patient’s estimated survival rate is 21–30 months (18–20). In principle, HCC is an indication for liver transplantation, which is able to provide a definitive cure to a subset of patients. However, eligibility for receiving organs from the UNOS or Eurotransplant registry is linked to the “Milan criteria” for HCC staging (one lesion ≤5 cm; alternatively, up to three lesions, each <3 cm; no extrahepatic manifestations; no evidence of gross vascular invasion) (21). The patient's HCC stage does not meet the Milan criteria because one nodule is larger than 3 cm; however, since his tumor has not progressed to gross vascular invasion, he would still have a fairly good oncologic survival chance if transplanted. His physicians see two potential options: related living donor liver transplantation or transplantation of a liver made up of the patient's cells from a chimeric pig. As a potential living donor, the brother of the patient is willing to donate a liver lobe. He is a healthy 42-year-old craftsman, has a family with three children, and runs a small workshop with seven employees.

An institutional ethics committee reviews the case and comes to the following conclusion: as the patient is not eligible for receiving an organ from a deceased donor but may have a considerable chance of a complete cure, the experimental humanized pig liver transplantation approach is endorsed. On the one hand, the double equipoise principle is challenged by the fact that the patient's HCC stage impairs his chance of overall survival, and the donor risks, such as prolonged recovery and long-term health issues with incapacity to work, are not easily justified in this scenario. On the other hand, the patient would benefit from a functional human liver without the need of immunosuppression. The committee therefore suggests the experimental approach as a first-line therapy and the living donor transplantation as a back-up strategy in the case of graft failure (initial non-function) of the humanized pig liver approach (the current status regarding chimeric liver transplantation using blastocyst complementation is reviewed in (22)).

## Applying our scenario in Germany as example

Returning to our fictitious patient with liver carcinoma, we will run the scenario as it could play out in the German jurisdiction with respect to the steps outlined in Figure 1.

The German research group will pursue the two-pronged approach, deciding to combine human iPSCs with a liver defective porcine embryo via blastocyst complementation. For an overview of potential applicable laws, see Table 1.

**Table 1:** Illustration of German laws as to the question of which framework could capture the chimeric pig, the human iPSCs, and the xeno-organ.

Entity	Legal framework	Reason it might apply	Obstacle/problem
Chimeric animal	National animal welfare law (TierSchG)	TierSchG ensures the protection of the lives and wellbeing of animals to ensure no pain, suffering, or harm is done without good reason. Chimeric animals should logically enjoy the same protection.	The breeding law as per TierSchG is not triggered. Norms aiming at the regulation of the use of human cells for breeding assume that a human ovum is the basis for the process.
	Embryo Protection Act (ESchG)	Contains provisions regarding changing human germline cells. Inserting human iPSCs into the pig blastocyst is creating a part-human embryo.	An embryo is defined as fertilized human egg or a totipotent cell removed from an embryo. The status of human material when inserted into an animal is unclear.
	German Technology Act (GenTG)	Regulates genetically modified organisms and includes genetically altered animals.	Unclear whether mixing cells from humans and animals is in scope. Unclear whether further use of the chimeric animal for xenotransplantation purposes would be permitted.
	Advanced Therapeutic Medicinal Product (ATMP) acc. to § 4b AMG and ATMP directive	… defines a medicinal product as a substance, which includes living animals, to be used in or on the human body.	Unclear whether the pig itself will be viewed as an MP for application on humans or just as a vessel for the actual ATMP.
Induced pluripotent stem cells (iPSCs)	ATMP acc. to § 4b AMG and ATMP directive	Applies	Opens the question of whether iPSCs will be viewed as a tissue-engineered product or a gene therapy medicinal product.
Xeno-organ	ATMP acc. to § 4b AMG and ATMP directive	Applies	Opens the question of whether xeno-organs will be viewed as a tissue-engineered product or a gene therapy medicinal product.

### Which framework captures the chimeric pig?

There are several laws in Germany that could potentially be applied but a general problem is that the introduction of iPSCs into a pig blastocyst to create a chimeric animal is under-regulated: norms aiming at the regulation of the use of human cells for breeding assume that a human ovum is the basis for the process and thus do not trigger breeding laws, as per the national animal welfare law (TierSchG). The process of creating the resulting chimeric entity, which is part animal and part human, is therefore not regulated by these norms. At the same time, even statutory documents such as the German Embryo Protection Act (ESchG), which for the reasons outlined does not, prima facie, seem to apply, sometimes contain provisions dealing with germline modification (§ 5 ESchG). It has been argued, though, that the ESchG, due to prohibition of analogy, cannot be applied to artificially created germ cells (23).

In theory, genetically modified organisms are captured by the European GMO directive and thus, in our case, the German genetic engineering law (GenTG), which describes them as “*an organism […] whose genetic material has been changed in a way […] which would not occur naturally*” (24). Indeed, the clarifying opinion by the Court of Justice of the European Union from 2018 seems to suggest classification of edited animals as genetically modified organisms (GMOs) and it thus seems to suggest classification of edited animals as GMOs (25) and it thus seems clear that multitransgenic pigs are captured by this law. However, it is unclear whether the law would apply to human–animal mixtures as well even though it allows mixing of cells from different organisms and adding foreign DNA.

An alternative route would be classification of the pig as an Advanced Therapy Medicinal Product (ATMP). The German Medicinal Product Act (AMG) defines medicinal products as substances, which, in turn, can be “*bodies of animals, including those of living animals*” (26). The question is, then, whether the chimeric pig itself can and should be classified as a medicinal product (MP).

Still, in light of the above, the health care professional (HCP) decides to file the request for approval for the creation of the chimeric pig as a GMO with the competent state authority since GMO approval lies with the state in Germany.

### What is the end product and by which regulation is it captured?

Unarguably, there are at least two components that need separate regulatory approval: on the one hand, the human iPSCs derived from the patient, which constitute an ATMP, more precisely, either a tissue-engineered product (TEP) or, if they are indeed genetically modified upfront, e.g., to knock out central nervous system contribution, they might be classified as a gene therapy medicinal product.

On the other hand, the creation of the chimeric pig needs to be approved as well, as described above. The key question here is whether the pig as a whole can and should be viewed as an entity that will be applied to humans. This is interdependent with the question of what it will be classified as in the first place. For example, the GenTG explicitly excludes the application of GMOs on humans. As the xenoliver will be transplanted into the patient, though, it seems intuitive that it will be viewed as a MP, regardless of what the pig is classified as, which means that it will very likely need a separate approval process as an ATMP, i.e., a tissue-engineered product or gene therapy medicinal product before transplantation.

Indeed, the Paul Ehrlich Institute had a prospective meeting regarding the regulatory classification of xenotransplantation products and decided that the AMG will be applied together with the ATMP directive (Directive (EG) Nr. 1394/2007); however, this discussion happened in the context of genetically edited, i.e., multitransgenic, pigs (27) and in practice has not been applied to a chimeric animal or its organ, respectively.

The treating physicians, therefore, file for approval for the generation of two more ATMPs with the Paul Ehrlich Institute: (1) the iPSCs from the patient and (2) the xenoliver.

### Who is the owner of the xenoproduct?

After these respective approvals have been granted, iPSCs are created from the patient's skin sample; any contribution to the central nervous system and the germ line has been knocked out. The patient signs a waiver of property rights to ensure that he has no claim to either the iPSC lines produced or the later resulting organs (step 1 in Figure 1). The HCPs decide that in order to maximize the chances of success, more than one pig should be generated. They therefore insert the iPSCs into five pig blastocysts, which are then transferred into a sow (step 2). After 114 days, four healthy piglets are born (step 3). Over the next 12–24 weeks, their livers are monitored through functional imaging as well as invasive diagnostics (step 4). After 24 weeks, the pigs and their livers, respectively, have grown enough to allow for transplantation, which succeeds at the first attempt (step 5). The livers from the four remaining pigs are not used at this point, as it is still an experimental approach. In future cases, though, it is conceivable that the unused organs from the pigs generated in excess would be distributed via the Eurotransplant system. The patient has to sign a waiver for liability claims (steps 6–8). Follow-up of the patient is analogous to human transplantation with extra “xenovigilance” in relation to the implanted organ (step 9).

## Results and discussion

With the high unmet medical need for organs, pig xenotransplantation could potentially cure millions of patients with life-threatening diseases. Recent advances in primate models as well as the first transplantations of xenogeneic organs into human recipients make clinical trials in the near future more likely. It is therefore necessary that regulatory authorities start to think about how such approaches would pan out in their respective jurisdictions. Our hypothetical case study elaborated how xenotransplantation could potentially play out in the current German regulatory situation. However, the three questions highlighted above have not been conclusively answered.

Regarding question 1 and which framework would apply, it seems to have been answered for multitransgenic pigs, but it is not at all clear whether this holds true for chimeric pigs.

In addition, question 2 regarding what the end product is might not end up being answered by simply splitting the “product” in several parts and treating them as separate entities from a regulatory standpoint. On top of that, since classification as an ATMP happens at the national level, ATMPs are not regulated concisely within Europe, with some states classifying biotechnologically altered tissue products as ATMPs and some as medicinal products.

Question 3 regarding who the owner of the xenoproduct is seems to be the most complex. Will there be a difference regarding patentability between human–animal chimeric and multitransgenic pigs? How will (non-) patentability influence downstream property rights? How will those in turn affect to whom the excess organs belong and how they will be distributed—if at all?

In summary, the example of Germany shows the fragmented nature of regulations governing human–animal chimeras for the purposes of xenotransplantation, which creates a technological context devoid of legal certainty. The moral charge of the subject matter, and the related intercultural divergence, might steer jurisdictions in different directions even within Europe. Where research takes place in countries with significantly different ethico-legal approaches, a common set of norms will be difficult to agree upon. A continued, systematic debate on common standards should therefore be a priority.

In addition, there are numerous ethical issues pertaining to xenotransplantation, which have been discussed for years (28–33) and fall into three broad categories: the first argues that certain scientific experiments should simply not be undertaken; the second warns of unforeseen consequences of genetically altering organisms; and the third pertains to the suffering of involved animals. In addition, there is a continuous and heated debate about the permissibility of mixing animal and human material in the academic (34–36) as well as the public sector (37–39).

These ethical and legal questions need to be addressed before such an approach ever becomes routine.
